# Experimental Determination of Molecular Weight-Dependent Miscibility of PBAT/PLA Blends

**DOI:** 10.3390/polym13213686

**Published:** 2021-10-26

**Authors:** Shen Su, Rodion Kopitzky, Christian Berrenrath

**Affiliations:** 1 Department of Circular and Bio-Based Plastics, Fraunhofer UMSICHT, Institute for Environment, Safety and Energy Technology, Osterfelder Str. 3, 46047 Oberhausen, Germany; Rodion.kopitzky@umsicht.fraunhofer.de (R.K.); christian.berrenrath@umsicht.fraunhofer.de (C.B.); 2 Department of Mechanical Engineering, Ruhr-University Bochum, Universitaet Str. 150, 44780 Bochum, Germany

**Keywords:** DSC, glass transition temperature *T_g_*, hydrolysis, melt blending, methanolysis, blend miscibility, number-average molecular weight *M_n_*, PBAT, SEM, solution blending

## Abstract

Blends of poly(butylene adipate-*co*-terephthalate) (PBAT) and polylactide (PLA) have attracted the attention of academia and industry as a sustainable material. Unfortunately, this combination results in problems related to poor miscibility on the molecular level. This study mainly aims to determine the influence of molecular weights on the miscibility of PBAT/PLA blends. First, polymers with various molecular weights were obtained by the hydrolysis of PBAT and methanolysis of PLA. Second, the two components were solution-blended with different molecular weights and weight ratios. Third, each blend was heated to the molten state and subsequently stored at room temperature. Finally, the samples were tested using DSC and SEM. The thermal analysis indicated that the difference in glass transition temperature between both components decreased from about 91 °C to 57 °C and 0 °C, as the number-average molecular weights (*M_n_*) decreased from 52/127 to 9.4/9 and 6.3/6.6 kg/mol. Moreover, the morphology changed from phase-separated with dispersed large particles gradually to uniform and homogeneous. This experimental work validated the trends predicted in the previous study, namely that PBAT/PLA blends changed the state from immiscible to partially miscible to fully miscible with decreasing *M_n_* values. Moreover, we discussed the influencing factors such as weight ratio, temperature, and molecular structure on the miscibility. Based on the results, this work contributes to developing partially miscible and compatible blends without additives.

## 1. Introduction

Bioplastics have been increasingly developed and utilized due to the UN Sustainable Development Goals [1], EU Bioeconomy Strategy [2], and the growing market demand [3]. The term “bioplastics” refers to either biodegradable or bio-based or both. Diverse bioplastics are currently available on the market. Among them, poly(butylene adipate-co-terephthalate) (PBAT) and polylactide (PLA) account for more than 10% of the global production capacity of bioplastics, respectively [3].

**Figure 1:**
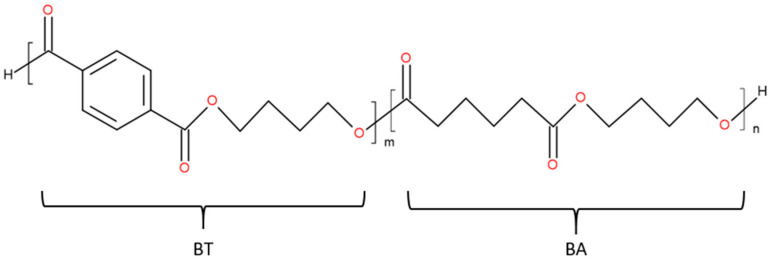
Chemical structure of PBAT.

PBAT is a petroleum-based or partly bio-based but biodegradable linear aliphatic-aromatic random copolyester composed of butylene terephthalate (BT) and butylene adipate (BA) [4,5] (Figure 1). BT is produced from the building blocks of terephthalic acid and 1,4-butanediol. BA is obtained by condensation of adipic acid and 1,4–butanediol. The molar ratio of the building blocks controls the polymer properties, processability, and biodegradability [5]. PBAT shows high toughness [6,7] but relatively low mechanical strength and high production costs [5].

**Figure 2:**
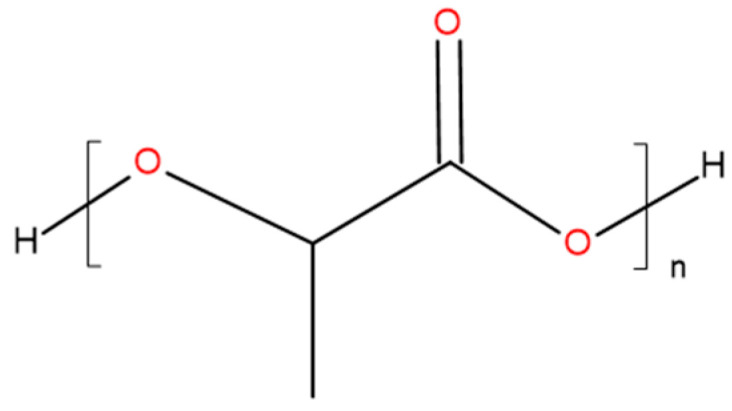
Chemical structure of PLA.

PLA is a linear aliphatic polyester produced through ring-opening polymerization of lactide [8,9] (Figure 2). PLA has a high modulus of elasticity, high strength, and easy processing [9,10], but brittleness at ambient conditions.

The drawbacks of individual polymer types are often compensable through mixing the polymers, a process known as blending. In recent years, many researchers have developed binary PBAT/PLA blends [11,12]. PBAT and PLA can be either melt blended [13] or solution blended [14]. Melt blending is applied to mix components at a temperature above their melting temperatures [15]. During the melt blending process, polymer degradation and intermolecular chemical reactions can occur. These reactions are avoidable by solution blending, including the dissolution of blend components in a suitable solvent, mechanical mixing, and solvent evaporation. However, solution blending is inappropriate for large-scale production due to the high cost of solvents and the difficulty of solvent evaporation [15].

A challenging problem of simple blending is the poor miscibility of unmodified PBAT/PLA blends. Before proceeding, clarification of the term “miscibility” is necessary. In this article, “miscibility” describes the behavior of a polymer blend by specifying the number of phases. The phase behavior is the first and most used criterion to evaluate the miscibility of polymer blends [16]. The commonly used methods for miscibility determination include DSC and SEM [17]. A binary polymer blend is miscible if it exhibits a homogeneous morphology with a single glass transition temperature (*T_g_*), which is between the *T_g_* values of the components [18]. A partially miscible binary blend presents some degree of homogeneity, where small amounts of each polymer in the blend can dissolve in the second polymer [15]; furthermore, it shows two *T_g_* values and each one shifts from the value of one component towards that of the other [17]. An immiscible binary blend exhibits phase separation [19] and two *T_g_* values, which are independent of blend compositions [17]. Compared with “miscibility”, “compatibility” defines the blend property profile from the practical perspective of a certain application. To increase the compatibility between the two components, various coupling agents or chemicals were applied. However, this research deals exclusively with the study of PBAT/PLA blend miscibility, which is determined experimentally.

Previous studies have determined whether PBAT and PLA are miscible with each other at different weight ratios and preparation methods. Farsetti et al. [20] reported that melt-blended PBAT/PLA blends with different compositions exhibit two-phase behavior and two separated almost unchanged glass transition temperatures (*T_g_*). The same phenomenon was confirmed by Su et al. [13], who melt blended these two commercially available products in a wide range of weight ratios. Liu et al. [14] found that with solution-blended material, the phase separation is more noticeable at the weight ratio (50/50) than (25/75) and (75/25). Gigante et al. [21] analyzed PBAT/PLA blends using SEM and found that the particle dimensions increased when the PBAT content increased the weight ratio between (90/10) and (75/25). Deng et al. [22] reported that melt-blended and compression-molded PBAT/PLA samples were immiscible based on the DSC results and that the blends formed *co*-continuous phase morphology according to the SEM analysis when the PBAT content was 20 wt.%. These miscibility studies revealed commercially available PBAT and PLA without variation in molecular weights were immiscible.

Two studies reported the dependence of the miscibility when one component has a high and a low molecular weight in the blend, respectively. Nofar et al. [12] used high and low molecular weight PLA for PBAT/PLA blends and found that the average diameter of the dispersed phase increased with increasing weight-average molecular weights of PLA. Dil et al. [23] melt blended PLA with high and low molecular weight PBAT and found the increase of molecular weight of PBAT reduces the miscibility in the PLA-rich phase dramatically. In addition to these experimental studies, Su [24] recently predicted the miscibility of PBAT/PLA blends concerning the molecular weights, composition, and temperature; blends tend to be more miscible (1) when the number-average molecular weights decrease; (2) when the PBAT weight fraction is <20 wt% or >80 wt%; and (3) when the blends are processed at a high temperature.

To the authors’ best knowledge, no study has reported the experimental results regarding the effect of the molecular weight of both components on the miscibility of PBAT/PLA blends. However, the knowledge of the molecular weight–dependent miscibility from the experimental research will contribute to the design of miscible and partially miscible PBAT/PLA blends without having to add any compatibilizers. This study aims to determine the effect of the molecular weight and blend ratio on the miscibility of PBAT/PLA blends experimentally. First, in the presence of a catalyst, PBAT was hydro–lyzed and PLA was methanolyzed to obtain polymers with different molecular weights, which were detected by GPC. Second, the two components with different molecular weights were solution blended at ratios of 80/20, 50/50, and 20/80. Third, these binary blends were heated up to the molten state and then stored at ambient temperature for four weeks. This was followed by a cryogenic fracture. Finally, to determine the miscibility, DSC and SEM were used. The experimental results from this research were compared with the previous prediction.

The novelty of this study is the experimental determination of the phase behavior of PBAT/PLA blends with different molecular weights of both components.

## 2. Materials and Methods

### 2.1. Materials

PBAT (Ecoflex F Blend C1200, BASF SE, Ludwigshafen, Germany [6]) possesses a density of 1.26 g/mol, a number-average molecular weight (*M_n_*) of 52.1 kg/mol, and a polydispersity index of 2.0.

PLA (Ingeo^TM^ Biopolymer 2003D, NatureWorks LLC, Minnetonka, MN, USA [25]) has a density of 1.24 g/mol, *M_n_* of 127.0 kg/mol, a polydispersity index of 1.6, and a D-isomer content of approximately 4.4%.

Sulfuric acid (96%), trichloromethane, ethanol, and methanol are purchased (Carl Roth GmbH + Co. KG, Karlsruhe, Germany) and used as received.

### 2.2. Preparation of Low Molecular Weight Polymers

PBAT and PLA granules were pre-dried in a vacuum oven (at 60 °C) before use. The preparation of low molecular weight polymers proceeds through catalytic hydrolysis of PBAT and catalytic methanolysis of PLA, respectively.

PBAT (10 g) was dissolved in trichloromethane (150 mL) under heating, reflux cooling, and stirring. A solution of sulfuric acid (0.2 mL) in distilled water (100 mL) was added to the PBAT solution. Then this mixed solution was heated at boiling temperature stirred under nitrogen supply and reflux cooling for a designated time. Subsequently, the solution was poured into cold ethanol (1000 mL), respectively. The precipitated PBAT was then obtained by filtering, washing with ethanol, and drying under the fume hood.

Similarly, the methanolysis of PLA is described as follows. First, PLA was dissolved in trichloromethane (150 mL) under heating, reflux cooling, and stirring. A solution of sulfuric acid (0.3 mL) and methanol (100 mL) was added to the PLA solution. Then this mixed solution was heated at boiling temperature and stirred under nitrogen supply and reflux cooling for a designated time. Subsequently, the solution was poured into cold ethanol (1000 mL). The precipitated PLA was obtained by filtering, washing with ethanol, and drying under the fume hood.

For simplicity, sample IDs for the polymer samples consist of a letter and a number, which are related to the polymer type and the degradation time (Table 1) For instance, “B0” means a PBAT sample without hydrolysis. “L4” means a PLA sample methanolyzed for 40 min.

### 2.3. Solution Blending

Before solution blending, the *M_n_* values of PBAT and PLA samples were investigated (for details, please see Section 2.5, Characterization). The blends were classified into three groups according to their *M_n_*:

High *M_n_* (PBAT) and high *M_n_* (PLA): B0L0;

Middle *M_n_* (PBAT) and middle *M_n_* (PLA): B4L10;

Low *M_n_* (PBAT) and low *M_n_* (PLA): B12L12.

Each group was prepared with three different weight ratios (80/20, 50/50, and 20/80). To achieve a total mass of 1.0 g, the two components were weighed separately according to the weight ratios. These two components were dissolved in trichloromethane (20 mL). Next, each polymer solution was poured into a Petri dish to evaporate trichloromethane under the fume hood. Then the samples were kept in a vacuum oven (60 °C, 2 h, and low vacuum) to remove trichloromethane. Finally, films of the polymer blends were obtained on the bottom of the Petri dishes. The formulations of the blends are listed in Table 2. For abbreviations, please refer to Table 1.

### 2.4. Melt Blending

Each polymer blend obtained by solution blending was filled into a crucible and heated to the molten state using a DSC device (DSC 204, F1 Phoenix^®^, Netzsch-Geraetebau GmbH, Selb, Germany). The heating conditions were 190 °C (exception: the heating temperature for B12L12 samples was 160 °C to avoid the thermal degradation) for 4 min under nitrogen supply (10 mL/min). After heating, the samples were stored at room temperature for four weeks.

### 2.5. Characterization

A GPC instrument (Agilent 1100 Series, PSS GmbH, Mainz, Germany) was applied to determine the molecular weights and weight distribution of the polymer samples. Polymethyl methacrylate (PMMA) standards with narrowly distributed molecular weights were employed to prepare the calibration curve. First, each polymer blend sample was weighed (9.0–9.5 mg) and dissolved in 1,1,1,3,3,3-hexafluoro-2-propanol (HFIP) (3 mL) containing potassium trifluoroacetate (KTFAc) (0.05 mol/L). The completely dissolved sample was then filtered through a PTFE-membrane filter (pore size: 0.45 µm). Each sample (100 µL) was injected into the mobile phase with an isocratic pump (G1310A, Agilent Series 1100) and flowed into the GPC columns (flow rate: 1 mL/min). The column set includes three columns connected in series (pore size: 1000 Å, 300 Å and 100 Å, 7 µm, 8.0 mm × 300 mm; PSS GmbH, Mainz, Germany). As the components left the columns, a refractive index (RI) detector (G1362, Agilent 1100 Series) was used to detect them. The software (WinGPC^®^ UniChrom, Version 8.3, PSS GmbH, Mainz, Germany) determined the number–average molecular weights (*M_n_*) and polydispersity index (PDI) of the samples.

An SEM instrument (Vega3, TESCAN ORSAY HOLDING a.s., Brno, Czech Republic) equipped with SE and BSE detectors was used to investigate the morphological properties. The samples were fractured under the cryogenic condition in liquid nitrogen. The fractured surfaces were sputter-coated with gold (120 s) and scanned at an accelerating voltage of 20 kV.

A DSC instrument (DSC 204 F1 Phoenix^®^, NETZSCH-Geraetebau GmbH, Selb, Germany) was used to determine the glass transition temperatures (*T_g_*) of the neat polymers and their blends. The DSC cell was constantly purged with nitrogen (flow rate: 20 mL/min). Each blend sample (approximately 10 mg) was sealed in an aluminum pan. The temperature program was set as follows: at the beginning, the sample was cooled from room temperature to −60 °C (cooling rate: 10 °C/min). After maintaining this temperature (−60 °C, 15 min), the first heating program started (from −60 to 190 °C, heating rate: 10 °C/min; exception: B12L12 samples were heated from −60 to 160 °C to avoid thermal degradation). After holding this temperature (2 min), the first cooling step was performed (from 190 to −60 °C, 10 °C/min). Subsequently, this temperature was maintained (–60 °C, 15 min). Finally, the second heating step (from −60 to 190 °C) was carried out with the same heating rate.

## 3. Results

### 3.1. Proposed Mechanisms of Hydrolysis and Methanolysis

**Figure 3:**
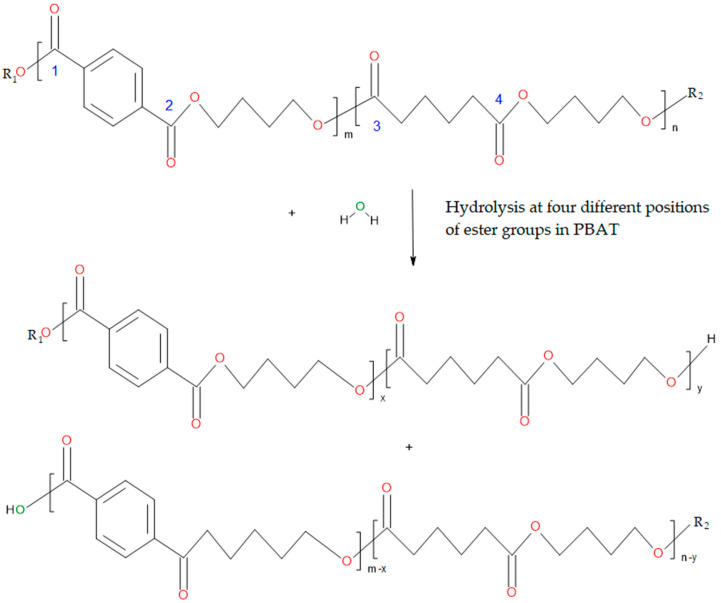
Proposed mechanism of the PBAT hydrolysis with four different reaction positions.

The hydrolysis of PBAT likely takes place randomly at the ester bonds along the backbone of the polymer. This proposed mechanism for PBAT hydrolysis is different from that described by Al-Itry et al. [26], which would only be suitable for di-block copolyesters of PBAT. Since this copolyester is usually composed of two randomly arranged monomers with three different building blocks, the hydrolysis results in the breakdown of PBAT into two OH–terminated PBAT molecules. The reaction with water molecules can occur at four different positions of the ester linkages (Figure 3).

Subsequently, the resulting PBAT molecules can further react with water molecules to generate smaller molecules. These products of hydrolysis may contain different ratios of monomers than the original polymer [24].

**Figure 4:**
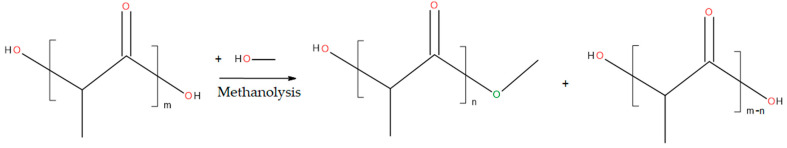
Proposed mechanism of the PLA methanolysis.

The methanolysis of PLA occurs randomly at the ester bonds along the polymer chain (Figure 4).

PLA consists only of lactic acid as a repeating unit. Methanolysis leads to a lower molecular weight PLA with a methyl group as an end group. The products obtained by the methanolysis can further react with methanol to generate smaller molecules.

### 3.2. Number-Average Molecular Weights and Polydispersity Index

**Figure 5:**
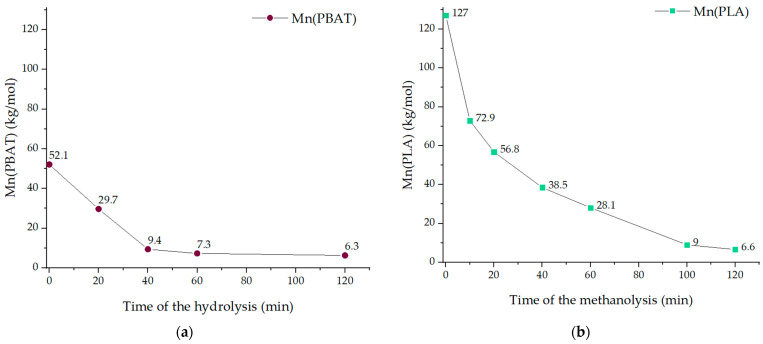
Number–average molecular weights (*M_n_*) of (**a**) PBATin dependence on the time of the hydrolysis and (**b**) PLA samples in dependence on the time of the methanolysis.

The catalytic hydrolysis of PBAT and catalytic methanolysis of PLA produced polymers with different *M_n_* and PDI. Their *M_n_* was determined using GPC (Figure 5).

With increasing reaction time from 0 to 120 min, *M_n_* (PBAT) decreased from 52.1 to 6.3 kg/mol and *M_n_* (PLA) decreased from 127 to 6.6 kg/mol. The two diagrams show that the rate of decrease in molecular weights tended to decrease as the reaction proceeded. The GPC equipment was not able to detect oligomers, which were increasingly formed during hydrolysis or methanolysis. Therefore, the real *M_n_* values of strongly hydrolyzed or methanolyzed samples are probably lower than the detected ones.

Moreover, the polydispersity index was determined. The PDI of the samples (B0, B4, B12, L0, L10, and L12) was in the range of 1.6–2.0.

### 3.3. Thermal Properties

The glass transition temperatures of neat PBAT, neat PLA, and PBAT/PLA blends were determined from the second heating scan. As seen in the DSC thermograph (Supplementary material: Figure S2), neat PBAT showed a *T_g_* of about –30 °C, while neat PLA exhibited a *T_g_* of approx. 60 °C.

The *T_g_* values of PBAT/PLA blends with different molecular weights and compositions are summarized in Table 3. These blends contained three different *M_n_* combinations, and each had three different weight ratios (80/20, 50/50, and 20/80), since both the molecular weights and the composition had influences on the blend miscibility according to the prediction [24].

For a better understanding of Table 3, some examples are given. B0L0 represents a blend consisting of the original polymers, having an *M_n_* (PBAT) of 52.1 kg/mol and an *M_n_* (PLA) of 127 kg/mol. The number 82 in the first line means that the weight ratio between PBAT and PLA is 80/20. The blend B0L0–82 represents the blend of B0L0 with a PBAT/PLA weight ratio of 80/20.

The high molecular weight blends B0L0–82, B0L0–55, and B0L0–28 exhibited two almost unchanged *T_g_* values (about −30 °C and 61 °C), which corresponded to the *T_g_* values of the neat components. The ∆*T_g_* values of these three samples were in the range of 90–93 °C, respectively.

The middle molecular weight blends B4L10–82, B4L10–55, and B4L10–28 had the *M_n_* (PBAT) = 9.4 kg/mol and *M_n_* (PLA) = 9.0 kg/mol obtained by hydrolysis and methanolysis. An exact specification of the *T_g_* (PLA) of B4L10-82 was not given due to the resolution. However, B4L10–55 and B4L10–28 indicated two separated but reduced ∆*T_g_* values of 57 and 56 °C, respectively, shifting into one another. This revealed improved miscibility between the two components.

The low molecular weight blends B12L12–82, B12L12–55, and B12L12–28, were made of strongly hydrolyzed and methanolyzed polymer components (*M_n_* (PBAT) = 6.3 kg/mol; *M_n_* (PLA) = 6.6 kg/mol). Only one single *T_g_* (−6 °C and −5 °C) was detected in the blends of B12L12-82 and B12L12-55, indicating good miscibility between the two components. For the blend B12L12-28, an exact specification of the *T_g_* from the second heating curve was not possible. However, its first heating curve also showed a single *T_g_* at about 44 °C.

It was concluded that the decrease in *M_n_* values tends to decrease the ∆*T_g_* of the samples, indicating that the PBAT/PLA blends reached the states from immiscible to partially miscible and completely miscible. The molecular weights affected the PBAT/PLA miscibility more than the weight ratio.

### 3.4. Morphological Properties

**Figure 6:**
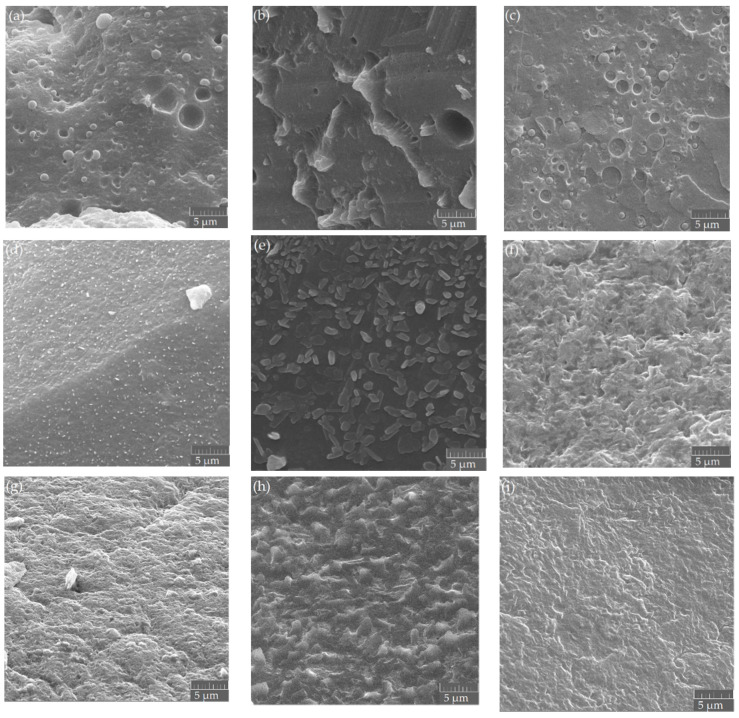
SEM micrographs of cryogenically fractured blend samples: (**a**) B0L0–82, (**b**) B0L0–55, (**c**) B0L0–28, (**d**) B4L10–82, (**e**) B4L10–55, (**f**) B4L0–28, (**g**) B12L12–82, (**h**) B12L12–55, (**i**) B12L12–28.

Cryogenically fractured surfaces of the samples are exhibited in Figure 6. Samples with the same *M_n_* combination are shown in one row and those with the same weight ratio are listed in one column. A scale (5 µm) was displayed in each SEM diagram.

The three blends of B0L0 [(a), (b), and (c)] showed phase-separated sea-island morphologies, as reported in previous studies [13]. The largest holes and most obvious unevenness on the surface were found in the blend B0L0–55, indicating that with the same *M_n_* values, the blend with the PBAT/PLA weight ratio of 50/50 was more difficult to mix than the blend with the composition of 80/20 or 20/80. This phenomenon is in agreement with the previous experimental study [13] and the prediction [24].

The B4L10 samples displayed that the morphology was phase-separated with distinctly reduced size of the dispersed particles. For the B12L12 samples, it was difficult to identify the phase boundaries of the PBAT and PLA. Therefore, the morphology of the blends tended to be more homogeneous as the molecular weights decreased. Moreover, with the same *M_n_* values, the phase separation was more noticeable at the weight ratio of 50/50 than at 80/20 and 20/80. These two tendencies determined experimentally are consistent with the tendencies predicted and simulated for PBAT/PLA blends [24].

For the three blends of B12L12, phase boundaries were difficult to see. The surfaces of B12L12–82 were generally homogeneous except for small fragments and small holes. The blend B12L12–55 showed slight unevenness due to the color difference. The blend B12L12–28 exhibited homogeneous surfaces overall.

It tended to show that the smaller the *M_n_* values of the blend components, the more homogeneous the cryogenically broken surface. For the same *M_n_* combination, the sample with the PBAT/PLA ratio of 80/20 or 20/80 seemed more homogeneous than the blend with the weight ratio of 50/50.

## 4. Discussion

### 4.1. Comparison of the Miscibility Results from the Prediction and the Tests

**Figure 7:**
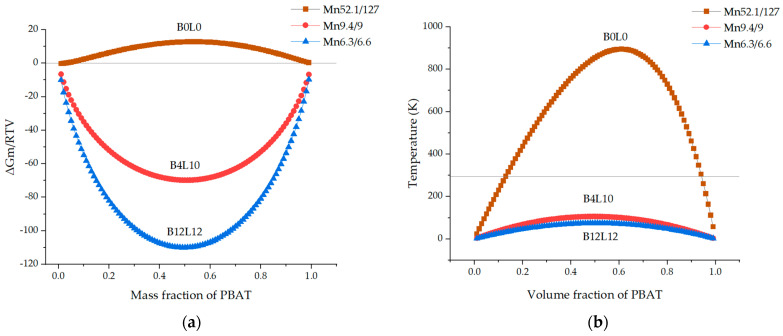
Miscibility simulation based on Su’s method [24]: (**a**) Phase diagram and (**b**) spinodal curves of PBAT/PLA blends: B0L0, B4L10, and B12L12 at 293 K.

The miscibility of a binary polymer blend depends on two necessary conditions [24]: (1) the negative free energy of mixing (∆*G_M_*) described by the equation ∆*G_M_* = ∆*H_M_* − *T*∆*S_M_*; and (2) the second derivative concerning the volume fraction (*Φ*) of the second blend components (∂^2^(∆*G_M_*)/∂*Φ*_i_^2^)*_T_*_,*p*_ > 0 [27]. Su [24] simulated the miscibility of PBAT/PLA blends using calculated solubility parameters, the Flory–Huggins model, and different molecular weights and weight ratios at room temperature. Based on his method, a phase diagram and the second derivative of ∆*G_M_* for the blends with the three different *M_n_* combinations are presented (Figure 7).

The phase diagram shows that the curve of B0L0 is overall positive, predicting immiscibility, which meets the experimental results with two almost unchanged *T_g_* values and phase separation in the morphology. Furthermore, it was observed that B0L0 samples were optically opaque (Supplementary Material Figure S2).

The curve of B12L12 is in the negative range in the phase diagram and lower than the line of spinodal decomposition on the right side, predicting good miscibility. This simulated result for B12L12 is in agreement with its experimental results, with the single *T_g_* detected in DSC and fine homogeneous morphology observed in SEM.

The curve of B4L10 is in the negative range but higher than that of B12L12 in the phase diagram. In the left diagram, B4L0 shows no spinodal decomposition at room temperature. Therefore, the two conditions for miscibility were satisfied. However, the DSC shows that B4L10 had two *T_g_* values, and each *T_g_* shifted from the value of one component towards that of the other. Furthermore, SEM presented a morphology with phase separation in B4L10. These results suggest that B4L10 is partially miscible. Two reasons may explain the difference between the prediction and experimental results. The first reason is the assumption that the polymers of one type have the same *M_n_* value. However, the molar masses are distributed. The PDI determined by GPC was 1.6–2.0. Thus, those polymer molecules possessing higher molecular weights could result in lower miscibility than expected. The second reason is that the molar ratio in PBAT was assumed to be 1:1 (BA:AT).

### 4.2. Factors Influencing The Miscibility

The variation of the molecular weights of both polymers (*M_n_* of 52.1/127, 9.4/9, and 6.3/6.6 kg/mol) exhibited a great influence on the miscibility. It was predicted that the lower the molecular weights, the better the miscibility of the blend [24]. This trend was validated by the experimental results in previous studies with different molecular weights of only one component [8,23] and in this study with the different *M_n_* values of both components. The PBAT/PLA blend B12L12 with the *M_n_* of 6.3 and 6.6 kg/mol showed good miscibility through its single glass transition temperature and relatively uniform morphology. The blend B4L10 with *M_n_* of 9.4 and 9 kg/mol showed partial miscibility with two glass transition temperatures shifting into each other and a fine morphology with small-dispersed particles. The partial miscibility of B4L10 may be due to some molecules with relatively higher molecular weights than the average *M_n_* values. The blend B0L0 exhibited very poor miscibility as mentioned above. This research focused exclusively on the miscibility between PBAT and PLA. However, miscibility and compatibility are not independent. Not only fully miscible but also partially miscible blends can be compatible. To achieve good mechanical properties, a certain degree of entanglements is necessary. Therefore, a blend with *M_n_* values of 10–20 kg/mol would probably achieve higher mechanical properties than a fully miscible blend with low *M_n_* values (around 6.5 kg/mol).

Weight ratios (80/20, 50/50, and 20/80) of the blend components exhibited some influences on the morphology but less influence on the difference of glass transition temperatures. The blends with the weight ratio of 80/20 or 20/80 showed more homogeneous cryogenically fractured surfaces than those with the weight ratio of 50/50 when they were not fully miscible. This observation is consistent with the previous prediction. Moreover, depending on the composition, a spinodal induced phase separation might affect the miscibility of a binary blend [28].

Temperature plays an important role in the preparation method of a polymer blend and its miscibility. In this study, PBAT and PLA were first solution blended at room temperature. In comparison, melt blending requires an elevated temperature (in this study, for blends with high and middle *M_n_*: 190 °C, and for the ones with low *M_n_*: 160 °C) so that both components reach the melt state while not thermally decomposing. According to the simulation, the higher the temperature, the better the miscibility of a PBAT/PLA blend [24]. The experimental analysis provides information about the current state of the system. In the molten state, a polymer blend could be the miscible blend. However, the molecules can gradually reach equilibrium, due to the segmental mobility, if the polymer blend is stored at a lower ambient temperature (above the *T_g_* (PBAT) ≥ −30 °C) for a sufficiently long time. The determination of the miscibility of a blend in the molten state would be only possible when the sample prepared in the molten state is quickly quenched and subsequently measured using DSC and SEM. The temperature change can lead to changes in the blend miscibility.

The PBAT structure is also crucial because its monomer ratio BA/AT affects the solubility parameter difference between PBAT and PLA. A BA−rich PBAT would be more miscible with PLA than an AT−rich PBAT [24].

## 5. Conclusions

PBAT and PLA with different molecular weights were obtained by catalytic hydrolysis and methanolysis. The *M_n_* values and PDI were determined using GPC. Then high, middle and low molecular weight PBAT/PLA were blended and then analyzed regarding the miscibility using DSC and SEM.

DSC analysis revealed that the difference of glass transition temperatures decreased from 91 to 57 and finally to 0 °C with decreasing *M_n_* values (from 52.1/127 to 9.4/9 and 6.3/6.6 kg/mol). The SEM showed that the morphology changed from phase-separated to fine and homogeneous, as the *M_n_* values decreased. For partially miscible and immiscible blends with the same *M_n_* values, the weight ratio of 80/20 and 20/80 resulted in smaller dispersed particles than that of 50/50.

The experimental results for B0L0 (immiscible) and B12L12 (immiscible) are in good agreement with the prediction. However, the blend B4L10 predicted to be miscible was experimentally determined as partially miscible. The reason for this difference is probably the assumption that a polymer sample possesses a uniformly *M_n_* value. In practice, the molecular weights are distributed. Therefore, those molecules having higher actual molecular weights than the average *M_n_* would have caused the partial miscibility of the blends. Furthermore, the weight ratio of the monomers in PBAT was assumed to be 1:1 (BA:AT), which could differ from the real molecular structure, affecting the accuracy of the prediction. The processing and storage temperature can affect miscibility. A PBAT/PLA blend can reach the miscible state more easily in the melt than at room temperature. The reason is that the segmental mobility of PBAT molecules is still high at room temperature, which is higher than the *T_g_* (PBAT) so that the blend system would reach equilibrium after a long time.

The miscibility of PBAT/PLA blends depending on the molecular weights of both components was experimentally determined in this study. This work also contributes to developing a partially miscible and compatible blend without additives.

## Figures and Tables

**Table 1:** Sample ID of (hydrolyzed) PBAT and (methanolyzed) PLA.

Sample ID	Polymer Type	Degradation Duration (min)
B0	PBAT	0
B2	PBAT	20
B4	PBAT	40
B6	PBAT	60
B12	PBAT	120
L0	PLA	0
L1	PLA	10
L2	PLA	20
L4	PLA	40
L6	PLA	60
L10	PLA	100
L12	PLA	120

**Table 2:** Formulation of solution–blended PBAT/PLA.

PBAT/PLA Blend ID	PBAT Component	PLA Component	PBAT Content (wt%)	PLA Content (wt%)
B0L0-82	B0	L0	80	20
B0L0-55	B0	L0	50	50
B0L0-28	B0	L0	20	80
B4L10-82	B4	L10	80	20
B4L10-55	B4	L10	50	50
B4L10-28	B4	L10	20	80
B12L12-82	B12	L12	80	20
B12L12-55	B12	L12	50	50
B12L12-28	B12	L12	20	80

**Table 3:** Glass transition temperatures of PBAT/PLA blends with different molecular weights and compositions.

Blend	*M_n_* (PBAT)/*M_n_* (PLA) (kg/mol)	82			55			28		
		*T_g_* (PBAT) (°C)	*T_g_* (PLA) (°C)	∆*T_g_* (°C)	*T_g_* (PBAT) (°C)	*T_g_* (PLA) (°C)	∆*T_g_* (°C)	*T_g_* (PBAT) (°C)	*T_g_* (PLA) (°C)	∆*T_g_* (°C)
B0L0	52.1/127	−32	61	93	−30	61	91	−29	61	90
B4L10	9.4/9	−31	**^1^	**^1^	−11	46	57	−37	18	56
B12L12	6.3/6.6	−6		0	−5		0	44 **^2^		0

Legend: ∆*T_g_*: difference of glass transition temperatures; **^1^: An exact specification of the *T_g_* (PLA) of B4L10 was not possible. **^2^: Data obtained from the first heating curve, since the *T_g_* was not able to be determined from the second heating curve.

## Data Availability

Further data in Supplementary Materials.

## Supplementary Materials

The following are available online at https://www.mdpi.com/article/10.3390/polym13213686/s1, Figure S1: DSC thermograms: the second heating curves of the neat PBAT, neat PLA, and the blends, Figure S2: Prepared blend B0L0-55 in a TGA crucible with a Fraunhofer employee card in the background.
