# Supplementary material for: Experimental Determination of Molecular Weight-Dependent Miscibility of PBAT/PLA Blends
Source: Polymers (Basel). 2021 Oct 26;13(21):3686. doi: 10.3390/polym13213686 (PMC8586918; doi:10.3390/polym13213686)
Supplement: Supplementary file 1 [file polymers-13-03686-s001.zip › polymers-1392693-supplementary.pdf]

Supplementary Material

Figure S1

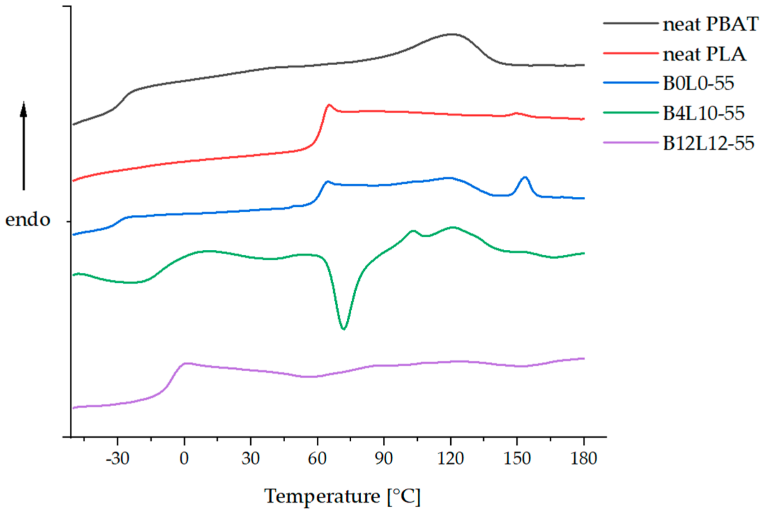

Figure S1. DSC thermograms: the second heating curves of the neat PBAT, neat PLA and the blends.

The software Netzsch Proteus Thermal Analysis was used to determine the T_g values.

Figure S2

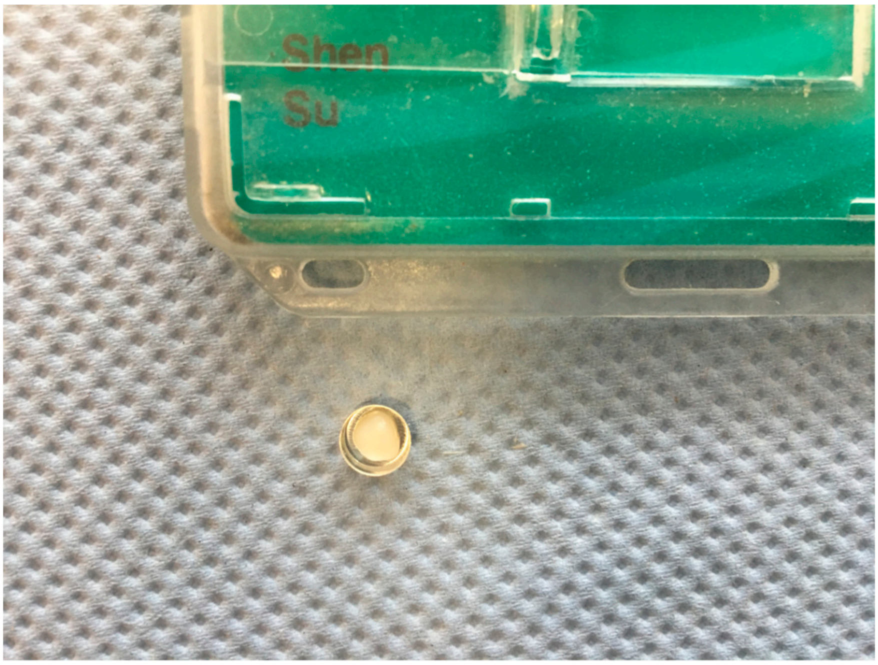

Figure S2. Prepared blend B0L0-55 in a TGA crucible with a Fraunhofer employee card in the background.
